# Linking language features to clinical symptoms and multimodal imaging in individuals at clinical high risk for psychosis

**DOI:** 10.1192/j.eurpsy.2020.73

**Published:** 2020-08-11

**Authors:** S. S. Haas, G. E. Doucet, S. Garg, S. N. Herrera, C. Sarac, Z. R. Bilgrami, R. B. Shaik, C. M. Corcoran

**Affiliations:** 1 Department of Psychiatry, Icahn School of Medicine at Mount Sinai, New York, New York, USA; 2 Boys Town National Research Hospital, Omaha, Nebraska, USA

**Keywords:** Clinical high risk for psychosis, multimodal, natural language processing, neuroimaging, sparse canonical correlation analysis

## Abstract

**Background.:**

Abnormalities in the semantic and syntactic organization of speech have been reported in individuals at clinical high-risk (CHR) for psychosis. The current study seeks to examine whether such abnormalities are associated with changes in brain structure and functional connectivity in CHR individuals.

**Methods.:**

Automated natural language processing analysis was applied to speech samples obtained from 46 CHR and 22 healthy individuals. Brain structural and resting-state functional imaging data were also acquired from all participants. Sparse canonical correlation analysis (sCCA) was used to ascertain patterns of covariation between linguistic features, clinical symptoms, and measures of brain morphometry and functional connectivity related to the language network.

**Results.:**

In CHR individuals, we found a significant mode of covariation between linguistic and clinical features (*r* = 0.73; *p* = 0.003), with negative symptoms and bizarre thinking covarying mostly with measures of syntactic complexity. In the entire sample, separate sCCAs identified a single mode of covariation linking linguistic features with brain morphometry (*r* = 0.65; *p* = 0.05) and resting-state network connectivity (*r* = 0.63; *p* = 0.01). In both models, semantic and syntactic features covaried with brain structural and functional connectivity measures of the language network. However, the contribution of diagnosis to both models was negligible.

**Conclusions.:**

Syntactic complexity appeared sensitive to prodromal symptoms in CHR individuals while the patterns of brain-language covariation seemed preserved. Further studies in larger samples are required to establish the reproducibility of these findings.

## Introduction

Schizophrenia is a major psychiatric disorder presenting with positive, negative, and cognitive symptoms [1]. Language disturbances are a cardinal feature of schizophrenia that manifest at all levels, from comprehension to production [2,3]. Language production disturbances have been reported in phonetics, morphology, syntax, semantics, and pragmatics; the most common abnormalities include idiosyncratic semantic associations, neologisms and word approximation, poverty of speech, and reduced grammatical complexity [2,4–7].

These language abnormalities implicate the corresponding brain networks. Current models of language processing support the dual-stream model, which specifies a ventral stream that primarily supports comprehension, and a dorsal stream that primarily supports articulation [8]. Typically, the ventral stream is largely bilateral while the dorsal stream is strongly left-lateralized [8]. Within the ventral stream, speech sounds are initially processed within the auditory regions of superior temporal gyrus while portions of the middle and inferior temporal lobe (including the fusiform gyrus) and the anterior temporal lobe correspond to the lexical interface, which links phonological to semantic information [9–11]. The dorsal stream includes Broca’s region in the inferior frontal gyrus, the insula, the parietotemporal sylvian region (considered a sensorimotor interface region), and motor and premotor cortical regions [9–11]. However, language does not simply involve language-specialized regions, but also relies on brain networks that support general cognitive functions [12,13], mainly the executive control network (ECN) [14], the salience network (SAL) [14], and the default-mode network (DMN) [15], which forms the functional basis of brain organization [16].

Multiple functional and structural neuroimaging studies in patients with schizophrenia [12,17–21], and in clinical and genetic high-risk groups [22–25], have established the presence of abnormalities in the language-related brain regions and in the networks supporting language-functions and their association with language dysfunction. By contrast, the corresponding literature in clinical high-risk (CHR) individuals is just beginning to emerge. Indicators of semantic dysfunction have been associated with lower gray matter density [26] and aberrant functional activation of brain regions within the language network [25].

Linguistic profiling has benefited from computational methods that enable the automatic and precise labeling of speech features in patients with schizophrenia and CHR [27–32]. Our group has demonstrated that semantic and syntactic abnormalities may be useful in predicting syndromal transition in CHR [27,28]; with measures of semantic coherence being the most discriminant.

In the current study, we extend our previous work, as we seek to relate automatically derived language features to brain structure and functional connectivity in CHR individuals. As language is supported by a wide range of regions and networks, we use a whole-brain, multivariate approach to our analysis. Specifically, we employ sparse canonical correlation analysis (sCCA) [33] to identify linked patterns of covariation between multiple linguistic features and brain morphology and functional connectivity. sCCA is an extension of traditional CCA, is more appropriate for smaller samples, it is less susceptible to overfitting and has been extensively used to describe brain-cognition associations by us [34,35] and others [36–38]. Our initial hypotheses are that (a) amount of speech and measures of syntactic complexity will show significant covariation with symptoms; (b) both syntactic and semantic features will covary with brain structural and functional measures of the language network and its functional integration with cognitive control networks; and (c) brain-language covariation patterns would be altered by CHR status.

## Methods

### Sample

Individuals at CHR for psychosis and healthy individuals (HIs) were recruited at Columbia University and at the Icahn School of Medicine at Mount Sinai (ISMMS), both in New York, USA. Individuals were characterized as CHR based on the Structured Interview for Prodromal Syndromes/Scale of Prodromal Symptoms (SIPS/SOPS) [39] if they met criteria for the attenuated positive symptom syndrome, which requires at least 1 SIPS/SOPS-positive item in the prodromal range (3–5) with symptoms beginning or worsening in the past year, and symptoms occurring at an average frequency of once per week in the prior month.

HIs had no personal history of any psychiatric disorders and no family history of psychosis in their first-degree relatives. Additionally, all participants were screened to exclude concomitant medical and neurological disorders, lifetime history of significant head trauma, current substance use disorders, contraindications to magnetic resonance imaging (MRI) scanning and were required to be fluent in English. Further details on recruitment and eligibility screening are presented in the Supplementary Material, Section 1.1.1. The sample included 46 CHR and 22 HIs (Table 1) of whom 30 (CHR = 17; HI = 13) were recruited at Columbia University and 38 at the ISMMS (CHR = 29; HI = 9) (Supplementary Material, Section 1.1.2 and Table S1).

**Table 1.** Demographic and clinical characteristics of the whole sample.

Variable	CHR individuals (*N* = 46)	Healthy individuals (*N* = 22)
Age (years)^a^	23.19 (5.09)	26.47 (3.65)
Sex (male/female)	22/24	13/9
Education (years)^a^	13.83 (2.23)	16.27 (1.55)
Handedness^a^		
Right (%)	44 (95.65%)	17 (77.27%)
Left (%)	1 (2.17%)	5 (22.73%)
Mixed (%)	1 (2.17%)	–
Antipsychotic medication use (%)	14 (30.43%)	–
SIPS/SOPS		
Total positive^a^	14.57 (3.45)	1.91 (2.29)
Total negative^a^	14.78 (6.71)	1.36 (1.56)
Total disorganized^a^	7.59 (4.55)	0.91 (1.31)
Total general^a^	10.63 (5.56)	1.41 (1.68)
GFS		
Role^a^	5.70 (2.05)	8.32 (0.95)
Social^a^	5.48 (1.49)	8.32 (0.95)

Continuous variables are shown as mean (standard deviation).

Abbreviations: CHR, clinical high risk; GFS, Global Functioning Scale; SIPS/SOPS, Structured Interview for Prodromal Syndromes/Scale of Prodromal Symptoms.

a Significant case–control differences at *p* < 0.05.

### Clinical assessment

In addition to the SIPS/SOPS, all participants were assessed using the Structured Clinical Interview for DSM-5 Axis 1 Disorders [40], Global Functioning Scale [41], and the Edinburgh Handedness Inventory [42] (Supplementary Material, Section 1.1.3).

### Language assessment

Naturalistic speech samples were obtained from all participants via open-ended 30–45 min narrative interviews following our previous work [27,28]. Interviews were transcribed by an independent Health Insurance Portability and Accountability Act (HIPAA) compliant company (https://sftp.transcribeme.com), and deidentified for analysis (Supplementary Material, Section 1.1.4). Interview transcripts were preprocessed as previously described [27,28] using the Natural Language Toolkit (NLTK; http://www.nltk.org/) [43]. The NLTK was used to extract the minimum, maximum, mean, and standard deviation of number of words per sentence to assess the amount of speech. Latent Semantic Analysis was used to quantify the minimum, maximum, mean, and standard deviation of the semantic coherence between consecutive sentences to assess semantics. Additionally, Part of Speech tagging based on the Penn Tree Bank was used with NLTK [43,44] to extract the frequencies of each tag in the speech specimens. Details of the process and definitions of the linguistic variables are provided in the Supplementary Material, Section 1.1.5 and Table S3.

### Neuroimaging

Structural and functional MRI were acquired on a GE MR750 3T scanner at Columbia University and on a 3T Siemens Skyra scanner (Erlangen, Germany) at the ISMMS. Both high-resolution structural and resting-state functional imaging data (rs-fMRI) were acquired in all participants using comparable protocols at each site as described in detail in the Supplementary Material, Section 1.2.1.

Following standard preprocessing and quality control, brain structural and rs-fMRI data were further analyzed to extract measures of brain morphometry and functional network connectivity (details in Supplementary Material, Sections 1.2.2 and 1.2.3). Segmentation and parcellation of the structural images were implemented in Freesurfer 6.0 (http://surfer.nmr.mgh.harvard.edu/) to yield 68 cortical thickness measures and 20 subcortical volume measures (defined in Supplementary Table S5). To enhance reproducibility, resting-state networks were defined using the templates available through the Functional Imaging in Neuropsychiatric Disorders Lab at Stanford University, USA (https://findlab.stanford.edu/functional_ROIs.html) for the language network (LAN), DMN, ECN, SAL, sensorimotor network (SMN), and auditory network (AN) networks [13] (Supplementary Figure S1). In each participant, Fisher Z-transformed Pearson’s correlation coefficients were used to compute network cohesiveness (i.e., average correlation of each voxel’s time series with every other voxel within each network). Network integration was computed as the correlation between the average time-series of each pair of networks. This process yielded 11 network connectivity measures (Supplementary Table S4). Prior to further analyses, imaging datasets were harmonized using ComBat [45], a Bayesian batch adjustment approach that accommodates the effect of site (https://github.com/Jfortin1/ComBatHarmonization).

### Statistical analyses

#### Conventional statistical analyses

Group differences in demographic, clinical, and linguistic features were analyzed using univariate and multivariate analyses; age, sex, and site were included as covariates when appropriate. Additional group-level analyses were undertaken to assess group differences in brain structure and functional connectivity that are described only in the Supplementary Material, Section 1.3. For all analyses, results are considered significant at *p* < 0.05 following Benjamini–Hochberg false-discovery-rate correction for multiple testing.

#### Sparse canonical correlation analyses

We implemented sCCA [33] in MatlabR2018b using an in-house script in accordance with our previously published work [34,35,46] to test the association between the linguistic, clinical, and neuroimaging data (details in Supplementary Material, Section 1.4). We considered four datasets; a nonimaging dataset comprising the clinical variables (Supplementary Table S2), a nonimaging dataset comprising the linguistic variables (Supplementary Table S3), a functional dataset comprising the functional network connectivity variables (Supplementary Table S4), and a structural dataset comprising the morphometric variables (Supplementary Table S5). All datasets were normalized by calculating *Z*-scores for each variable prior to entering the sCCA. We conducted separate sCCAs, using identical procedures, to identify patterns of covariation between the language and clinical datasets in CHR-individuals only, and between the language dataset and each of the imaging datasets in all participants. Diagnostic group was included in the language dataset for each of the imaging sCCAs in order to examine group effects. Brain morphometry and functional connectivity were considered separately because they represent different aspects of brain organization (an analysis of the pooled imaging data is also presented in the Supplementary Material). For each sCCA, we selected the optimal sparse criteria based on the parameters that maximized the sCCA correlation. We then computed the optimal sCCA model and determined its significance using permutations (*n* = 10,000). The *p*-value was defined as the number of permutations that resulted in a higher correlation than the original data divided by the total number of permutations. Thus, the *p*-value is explicitly corrected for multiple testing as it is compared against the null distribution of maximal correlation values across all estimated sCCAs. In each sCCA model, each canonical variate relates a weighted set of linguistic features to a weighted set of imaging measures. The weights of each feature in each variate provide an indication of their importance in the model.

## Results

### Linguistic features in the cohort

Four linguistic features (foreign word, list item marker, plural proper noun, possessive wh-pronoun) were not included in subsequent analyses because more than 50% of the sample achieved the same score. The descriptive statistics of the remaining 38 linguistic features in each group are presented in Table 2 (Supplementary Table S6). No group differences were identified at *P*_FDR_ < 0.05 for the individual linguistic features.

**Table 2.** Linguistic features of the sample.

Language variable	CHR individuals (*N* = 46)	Healthy individuals (*N* = 22)
Volume of speech		
Sentence length, mean	13.52 (2.85)	13.14 (3.92)
Sentence length, standard deviation	10.17 (2.58)	10.05 (2.89)
Sentence length, maximum	63.43 (18.11)	66.86 (15.6)
Semantic properties		
Semantic coherence, mean	0.81 (0.04)	0.79 (0.05)
Semantic coherence, standard deviation	0.18 (0.03)	0.19 (0.03)
Semantic coherence, minimum	0.02 (0.18)	−0.03 (0.17)
Semantic coherence, maximum	0.99 (0.01)	1 (0.01)
Parts of speech-tagging		
Coordinating conjunction	0.03 (0.01)	0.03 (0.01)
Cardinal number	0.01 (0.003)	0.01 (0.003)
Determiner	0.06 (0.01)	0.06 (0.01)
Existential *there*	0.002 (0.001)	0.003 (0.001)
Preposition or subordinating conjunction	0.07 (0.01)	0.07 (0.01)
Adjective	0.04 (0.01)	0.04 (0.01)
Adjective, comparative	0.002 (0.002)	0.002 (0.001)
Adjective, superlative	0.001 (0.001)	0.001 (0.001)
Modal verb	0.01 (0.004)	0.01 (0.003)
Noun, singular or mass	0.08 (0.01)	0.09 (0.01)
Noun, plural	0.02 (0.01)	0.02 (0.01)
Proper noun, singular	0.02 (0.01)	0.02 (0.01)
Predeterminer	0.001 (0.001)	0.001 (0.001)
Possessive ending	0.0003 (0.0003)	0.0005 (0.0005)
Personal pronoun	0.11 (0.01)	0.1 (0.01)
Possessive pronoun	0.01 (0.003)	0.01 (0.003)
Adverb	0.09 (0.01)	0.08 (0.01)
Adverb, comparative	0.002 (0.001)	0.001 (0.001)
Adverb, superlative	0.0002 (0.0003)	0.0002 (0.0002)
Particle	0.004 (0.001)	0.004 (0.001)
“*To*”	0.02 (0.004)	0.02 (0.003)
Interjection	0.04 (0.02)	0.05 (0.03)
Verb, base form	0.04 (0.01)	0.03 (0.01)
Verb, past tense	0.03 (0.01)	0.03 (0.01)
Verb, gerund or present participle	0.02 (0.005)	0.02 (0.004)
Verb, past participle	0.01 (0.003)	0.01 (0.003)
Verb, non-third person singular present	0.05 (0.013)	0.04 (0.01)
Verb, third person singular present	0.03 (0.01)	0.03 (0.01)
Wh-determiner	0.003 (0.002)	0.003 (0.001)
Wh-pronoun	0.01 (0.003)	0.01 (0.002)
Wh-adverb	0.01 (0.002)	0.01 (0.002)

Variables are shown as mean (standard deviation).

Abbreviation: CHR, clinical high risk; detailed definition of each variable is provided in Supplementary Table S3.

### Linked dimensions of language and clinical symptoms

**Figure 1.**
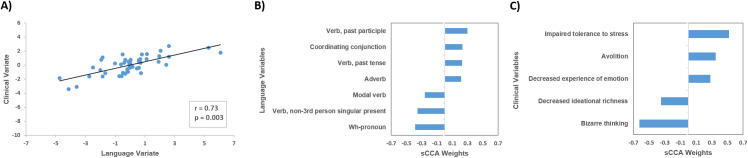
Sparse canonical correlation analysis (sCCA) for language features and clinical symptoms in individuals at clinical high-risk (CHR) for psychosis. (A) sCCA of linguistic features and clinical symptoms in CHR individuals identified a single significant mode; (B) linguistic features with the highest absolute weights; and (C) clinical symptoms with the highest absolute weights. Additional information in Supplementary Tables S7 and S8.

This sCCA model identified a single significant mode (canonical *r* = 0.73; *p* = 0.003) (Figure 1A). The weights of all the variables examined are provided in Supplementary Tables S7 and S8. The most heavily weighted linguistic features involved measures of syntactic complexity: use of coordinating conjunctions, adverbs, and verbs (Figure 1B). The most heavily weighted clinical measures were avolition, decreased experience of emotion, impaired tolerance to stress, bizarre thinking, and decreased ideational richness (Figure 1C).

### Linked dimensions of language and functional connectivity

**Figure 2.**
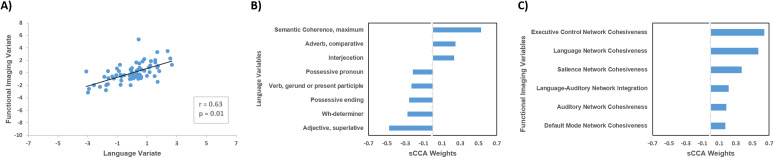
Sparse canonical correlation analysis (sCCA) for language features and resting-state network functional connectivity in the entire study sample. (A) sCCA of linguistic features and resting-state network functional connectivity in the entire sample identified a single significant mode. The weight of diagnosis was −0.03; (B) linguistic features with the highest absolute weights; and (C) connectivity measures with the highest absolute weights. Additional information in Supplementary Tables S9 and S10.

This sCCA model identified a single significant mode (canonical *r* = 0.63; *p* = 0.01) (Figure 2A); no further modes were significant (unadjusted *p* > 0.05). The weights of all the variables examined are provided in Supplementary Tables S9 and S10. We found negligible effects of diagnosis (weight = −0.03) and handedness (weight = 0). The most heavily weighted linguistic features involved measures of coherence (maximum semantic coherence) and measures of syntactic complexity involving the use of adjective, determiners, verbs, and pronouns (Figure 2B). The most heavily weighted connectivity measures were cohesiveness of the LAN, ECN, SAL, AN, and DMN networks and integration between the LAN and AN networks (Figure 2C).

### Linked dimensions of language and brain structure

**Figure 3.**
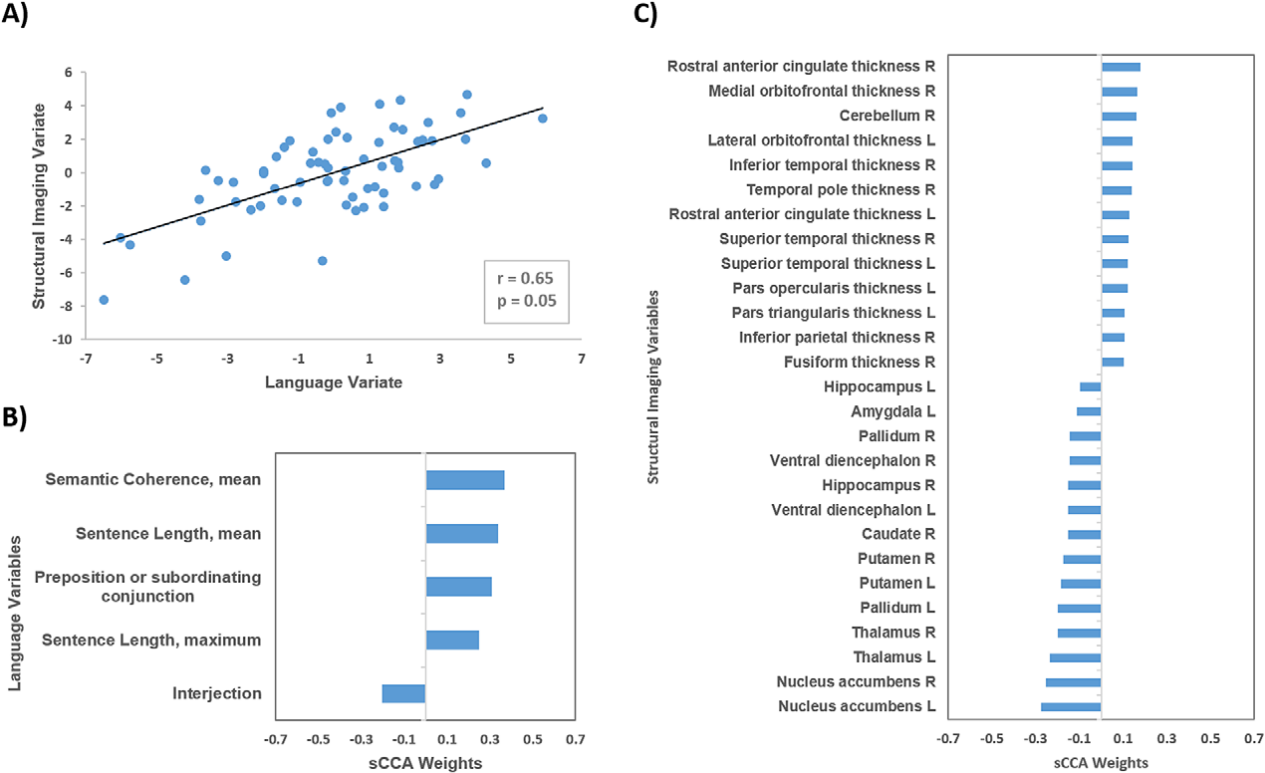
Sparse canonical correlation analysis (sCCA) for language features and brain morphometry in the entire study sample. (A) sCCA of linguistic features and brain structure in the entire sample identified a single significant mode. The weight of diagnosis was negligible (*w* = 0.07); (B) linguistic features with the highest absolute weights; and (C) brain morphometry measures with the highest absolute weights. Additional information in Supplementary Tables S11 and S12.

This sCCA model identified a single significant mode (canonical *r* = 0.65; *p* = 0.05); no further modes were significant (unadjusted *p* > 0.05) (Figure 3A). The weights of all the variables examined are provided in Supplementary Tables S11 and S12. The contribution of diagnosis (weight = 0.07) and handedness (weight = 0.02) were negligible. The most heavily weighted linguistic features involved measures of volume of speech relating to sentence length, mean semantic coherence, and measures of syntactic complexity (interjection and subordinating conjunction) (Figure 3B). The weights for the cortical and subcortical features were generally low (range of absolute values: 0.01–0.25). Amongst cortical regions with the highest values observed were left pars opercularis and triangularis, the bilateral superior temporal gyrus and rostral anterior cingulate and on the right, the medial orbitofrontal gyrus and frontal pole, the temporal pole and the inferior temporal and fusiform gyri, and inferior parietal lobule (Figure 3C). A large number of subcortical regions negatively covaried with linguistic features including bilateral thalamus, hippocampus, nucleus accumbens, pallidum and ventral diencephalon, the right amygdala, and left caudate nucleus.

## Discussion

We found no effect of diagnosis on individual linguistic features obtained in CHR and HIs. Nevertheless, in the CHR individuals, measures of syntactic complexity covaried with negative symptoms reflecting avolition and poverty of thought and disorganized symptoms of bizarre thinking. Linguistic measures of the amount of speech, semantic coherence, and syntactic complexity covaried with the measures of brain structure and resting-state functional connectivity that emphasized regions and networks involved in speech and language processing. There was no diagnostic effect on these patterns of covariation, although the study may not have been sufficiently powered to detect such a difference.

### Language features and clinical symptoms

In an independent sample of 34 CHR individuals, we have previously reported associations of the SIPS/SOPS total negative symptom severity with maximum phrase length, minimum semantic coherence, and use of determiners, respectively, implicating the amount and the semantic and syntactic organization of speech [27]. In our subsequent study involving 93 CHR individuals, no association between language features and clinical symptomatology was identified [28]. Here we found that predominantly negative symptoms covaried mainly with measures of syntactic complexity. One interpretation is that subtle disturbances in syntactic complexity may be more sensitive to clinical symptoms than other linguistic features. Alternatively, linguistic-clinical associations may depend on the specific characteristics of the CHR sample and should be further investigated across samples.

### Language and functional connectivity

The pattern of covariation between language features and resting-state connectivity was comparable in CHR and HIs. As expected, the cohesiveness of the LAN network emerged as one of the variables most strongly associated with language features. The cohesiveness of the DMN in connection to language is supported by prior studies linking introspective functions with verbal resources [47]. Efficient language and speech processing depend on multiple other cognitive functions, notably attention/salience, working memory, and cognitive control [48]. Aligned with this notion, the sCCA results underscore the importance of the cohesiveness of the ECN for language, which supports goal-directed behaviors [49] and is modulated by general cognitive effort [50]. The ECN and SAL networks are considered dissociable respectively supporting sustained and adaptive cognitive control [51]. In the context of the current results, the role of the ECN involves the moment-to-moment monitoring of speech production while SAL connectivity may be relevant to the adaptive control of semantic and syntactic organization of speech as dictated by contextual demands. We note the positive weight of the integration between LAN and AN for language processing. Studies of patients with schizophrenia have repeatedly found that the functional integration of these two networks is reduced but this feature may mainly arise in the context of hallucinations [52]. Generally, the absence of an effect of CHR status in this analysis suggests that this effect is either too small to be detected in the current sample or that the mapping of linguistic features to the resting-state connectivity is not disturbed in CHR individuals.

### Language features and brain structure

The sCCA links semantic coherence and syntactic complexity with variation mainly in prefrontal, and temporal regions and subcortical volumes. It is currently thought that semantic processing in the brain follows a “spoke-and-hub” model [53,54]; modality-specific content, primarily involving attributes, is represented in the spokes while more-abstract, amodal representations, referring mainly to semantic significance, are held in the hubs. Further, modality-specific representations are mainly left-lateralized for verbal content and right-lateralized for visual content while amodal representations are distributed in hubs in both hemispheres. Although there is debate about the number and locations of semantic hubs, plausible candidates have been proposed in different cortical regions, including the anterior-inferior-temporal lobe [54,55] the anterior-inferior-parietal [56] and the inferior-frontal cortex [57–61]. Of note, the top weighted regions, map closely to temporal and prefrontal sematic hubs. The neural correlates of syntactic processing are also debated [62] but there is general agreement that the key regions involved are the left opercular and left triangular portions of the inferior frontal cortex [63]. Our results therefore conform to current expectations regarding brain structure-language mapping. No effect of group was detected in this analysis which may relate to issues of power or may indicate that the mapping of linguistic features to brain structural measures is not disturbed in CHR individuals.

### Limitations

The main limitation of the current study is the small sample size particularly with regards to HIs. Additionally, site effects may have further influenced the power of the study to detect diagnostic differences. We therefore consider our data preliminary pending replication in larger samples. The linguistic and neuroimaging features examined were chosen for their potential translational value because of the relative ease in collecting such data in clinical settings. Brain structural and resting-state data acquisition have the advantage of brevity and does not require active patient engagement. Similarly, linguistic features were selected according to our prior data and were based on free natural speech which is easy to elicit in clinical settings. Future studies could expand the range of features to include speech graphs [64], prosody, pragmatics, metaphoricity [65], and discourse or conversations. Some CHR individuals were prescribed antipsychotics at the time of testing although their cumulative exposure was minimal. Nevertheless, an effect of medication cannot be conclusively excluded.

## Conclusion

Overall, we identified significant patterns of covariation between linguistic features and clinical symptoms in CHR individuals. The linguistic features were predominantly linked with negative symptoms and bizarre thinking, suggesting these symptoms co-occur with alterations in language processing in CHR. Future studies will be necessary to determine whether symptoms and language processing changes emerge simultaneously, or the onset of one precedes the other. No diagnostic effect was noted in the pattern of covariation between linguistic features and brain morphometry and resting-state network connectivity. These findings suggest relatively intact patterns of brain-language covariance. Further studies are needed to confirm the reproducibility of these findings.

## Data Availability

The data are available upon request from the National Data Archive and from Dr. Corcoran.

## Supplementary material

For supplementary material accompanying this paper visit http://dx.doi.org/10.1192/j.eurpsy.2020.73. click here to view supplementary material
