# Supplementary material for: Linking language features to clinical symptoms and multimodal imaging in individuals at clinical high risk for psychosis
Source: Eur Psychiatry. 2020 Aug 11;63(1):e72. doi: 10.1192/j.eurpsy.2020.73 (PMC7443790; doi:10.1192/j.eurpsy.2020.73)
Supplement: Supplementary file 1 [file S0924933820000735sup001.docx]

**Linking language features to clinical symptoms and multimodal imaging in individuals at clinical high risk for psychosis**

**Supplementary Material 1. Supplementary Methods**

**1.1 Sample**

1.1.1 Recruitment

1.1.2 Sample characteristics by site

Supplementary Table S1. Demographic and clinical characteristics of the sample at each site

1.1.3 Clinical assessment

Supplementary Table S2. Definition of clinical features assessed

1.1.4 Language assessment

1.1.5 Speech processing

Supplementary Table S3. Definition of the linguistic features assessed

1.1.6 Speech analyses

1.1.7 Code availability

**1.2. Neuroimaging**

1.2.1 Neuroimaging acquisition

1.2.2 Resting-state functional MRI data processing

Supplementary Figure S1. Spatial definition of the resting-state networks

Supplementary Table S4. Definition of functional connectivity variables

1.2.3 Structural MRI data processing

Supplementary Table S5. Definition of morphometric variables derived via Freesurfer 6

**1.3 Case-control differences in brain morphometry and resting-state connectivity**

**1.4 Sparse canonical correlation analysis (sCCA)**

**2. Supplementary Results**

**2.1 Language**

Supplementary Table S6. Linguistic features of the sample at each site

**2.2 sCCA for language and clinical features in the CHR sample**

Supplementary Table S7. Weights of the linguistic features in the significant mode of the sparse canonical correlation of linguistic and clinical measures in the CHR sample

Supplementary Table S8. Weights of the clinical features in the significant mode of the sparse canonical correlation of linguistic and clinical measures in the CHR sample

**2.3 sCCA for language and resting-state network connectivity in the whole sample**

Supplementary Table S9. Weights of the linguistic features in the significant mode of the sparse canonical correlation of linguistic and brain functional measures in the whole sample

Supplementary Table S10. Weights of the functional features in the significant mode of the sparse canonical correlation of linguistic and brain functional measures in the whole sample

**2.4 sCCA for language and brain structure** **in the whole sample**

Supplementary Table S11. Weights of the linguistic features in the significant mode of the sparse canonical correlation of linguistic and brain structural measures in the whole sample

Supplementary Table S12. Weights of the morphometric features in the significant mode of the sparse canonical correlation of linguistic and brain structural measures in the whole sample

**2.5 sCCA for language and pooled structural and functional neuroimaging variables**

**2.6 Case-Control differences in morphometry and functional connectivity**

**1. Supplementary Methods**

- 1. **Sample**
     1. **Recruitment**

All participants, regardless of diagnosis, were recruited into the study via local distribution of flyers and online advertisements in the New York City Metropolitan area, and referrals from schools, brochures, the internet, other programs, and the community. The process was the same at both recruitment sites.

For individuals at clinical high risk for psychosis, inclusion criteria were based on meeting criteria for Attenuated Positive Syndrome as described in the main text. Healthy individuals were selected to be free of personal lifetime history of psychiatric disorders based on the Structured Clinical Interview (SCID) for DSM-5^1^ and absence of family history in first degree relatives. Adopted individuals were thus excluded.

Further eligibility criteria for all participants were: (a) age 14-35 years; (c) no imminent risk of harm to self or others; (d) no past or current substance use disorder; (c) no major medical or neurological disorder; (e) no intellectual disability (IQ < 70); (f) no vision deficits not amenable to correction by wearing glasses; (g) no hearing deficits; (h) no contraindication to MRI (e.g., metal implants or paramagnetic objects. Additional exclusion criteria for women were pregnancy or nursing.

**1.1.2 Sample characteristics by site**

Patients and healthy participants were recruited at Columbia University, and at the Icahn School of Medicine at Mount Sinai (ISMMS) in New York. The same eligibility criteria were followed at both sites.

| **Supplementary Table S1. Demographic and clinical characteristics of the sample recruited at each site** | | | | |
| --- | --- | --- | --- | --- |
|  | **Columbia University** | | **ISMMS** | |
| **Variable** | **CHR**  **Individuals**  **N=17** | **Healthy**  **individuals**  **N=13** | **CHR Individuals**  **N=29** | **Healthy individuals**  **N=9** |
| **Age (years)**^1^ | 22.43 (4.53) | 26.03 (3.96) | 23.63 (5.42) | 27.12 (3.26) |
| **Sex (Male/Female)** | 8/9 | 9/4 | 14/15 | 4/5 |
| **Education (years)**^1^ | 13.18 (2.24) | 15.92 (1.71) | 14.21 (2.18) | 16.78 (1.2) |
| **Handedness**^2^ |  |  |  |  |
| **Right (%)** | 16 (94.12%) | 11 (84.62%) | 28 (96.55%) | 6 (66.67%) |
| **Left (%)** | 0 | 2 (15.38%) | 1 (3.45%) | 3 (33.33%) |
| **Mixed (%)** | 1 (5.88%) | 0 | 0 | 0 |
| **Antipsychotic medication use (%)** | 8 (47.06%) | - | 6 (20.69%) | - |
| **SIPS/SOPS** |  |  |  |  |
| **Total Positive Symptoms**^1,2,4^ | 16.35 (2.67) | 2.62 (2.72) | 13.52 (3.46) | 0.89 (0.78) |
| P1 – Unusual  Thought  content^1,2,3^ | 4.00 (0.87) | 0.54 (0.88) | 3.45 (0.63) | 0.11 (0.33) |
| P2 –  Suspiciousness^1,2^ | 3.71 (0.85) | 0.54 (0.88) | 3.28 (1.13) | 0 |
| P3 –  Grandiosity^1^ | 2 (1.66) | 0.31 (0.48) | 1.17 (1.42) | 0.22 (0.44) |
| P4 – Perceptual  abnormalities^1,2,3^ | 3.88 (0.33) | 0.69 (1.11) | 3.17 (1.39) | 0.33 (0.71) |
| P5 – Disorganized communication^1,2^ | 2.77 (0.66) | 0.54 (0.66) | 2.45 (1.15) | 0.22 (0.67) |
| **Total Negative Symptoms**^1,2,3^ | 18.94 (5.46) | 1.69 (1.55) | 12.35 (6.22) | 0.89 (1.54) |
| N1 – Social  isolation or  withdrawal^1,2^ | 3.35 (1.22) | 0.31 (0.63) | 2.59 (1.38) | 0.33 (0.5) |
| N2 – Avolition^1,2,3^ | 3.88 (1.54) | 0.15 (0.38) | 2.03 (1.24) | 0.11 (0.33) |
| N3 – Decreased  expression of  emotion^1,2^ | 1.88 (1.17) | 0.46 (0.97) | 1.69 (1.28) | 0.11 (0.33) |
| N4 – Decreased  experience of  emotion^1,2^ | 3.29 (1.83) | 0 | 2.62 (1.57) | 0 |
| N5 – Decreased  ideational  richness^1,2,3^ | 2 (1.27) | 0.69 (0.63) | 1.21 (1.21) | 0.22 (0.67) |
| N6 –  Deterioration of  occupational functioning^1,2,3^ | 4.53 (1.5) | 0.08 (0.28) | 2.21 (1.92) | 0.11 (0.33) |
| **Total Disorganization Symptoms**^3^ | 12.18 (2.77) | 1.31 (1.49) | 4.9 (2.93) | 0.33 (0.71) |
| D1 – Odd  behavior or  appearance^1,2,3^ | 3.00 (1.12) | 0.69 (0.95) | 1.00 (1.04) | 0.22 (0.44) |
| D2 – Bizarre  thinking^1,2,3^ | 3.41 (0.62) | 0.08 (0.28) | 1.1 (1.14) | 0.11 (0.33) |
| D3 – Trouble with  focus and  attention^1,2,3^ | 3.77 (1.03) | 0.31 (0.63) | 2.03 (1.4) | 0 |
| D4 – Impairment  in personal  hygiene^1,3^ | 2 (1.77) | 0.23 (0.44) | 0.76 (1.3) | 0 |
| **Total General Symptoms**^1,2,3^ | 14.88 (4.09) | 1.85 (1.99) | 8.14 (4.76) | 0.78 (0.83) |
| G1 – Sleep  disturbance^1,2,3^ | 3.59 (1.46) | 0.46 (1.13) | 1.69 (1.61) | 0.33 (0.71) |
| G2 – Dysphoric  mood^1,2,3^ | 4.82 (1.38) | 0.54 (0.78) | 3.1 (1.47) | 0.33 (0.5) |
| G3 – Motor  disturbance^1,2,3^ | 1.88 (1.45) | 0.46 (0.88) | 0.93 (0.88) | 0.11 (0.33) |
| G4 – impaired  tolerance to  stress^1,2,3^ | 4.59 (1.84) | 0.38 (0.65) | 2.41 (1.92) | 0 |
| **GFS** |  |  |  |  |
| **Role**^1,2^ | 5.06 (1.71) | 8.23 (0.93) | 6.07 (2.17) | 8.44 (1.01) |
| **Social**^1,2^ | 5.18 (1.47) | 8.31 (1.03) | 5.66 (1.49) | 8.33 (0.87) |
| Continuous variables are shown as mean (standard deviation); CHR=Clinical high risk; SIPS/SOPS = Structured Interview for Prodromal Syndromes/Scale of Prodromal Symptoms; GFS = Global Functioning Scale. ^1^significant case-control differences at Columbia at p<0.05 uncorrected; ^2^significant case-control differences at ISMMS; ^3^site differences in healthy individuals at p<0.05 uncorrected; ^4^site differences in CHR individuals at p<0.05 uncorrected | | | | |

**1.1.3. Clinical assessment**

Participants were initially screened by phone. Potentially eligible participants were assessed in person to confirm eligibility. Clinical assessments were undertaken by certified research assistants and were reviewed by the principal investigator. The Structured Interview for Prodromal Syndromes/Scale of Prodromal Symptoms (SIPS/SOPS)^2^ was used to assess positive, negative, disorganized, and general symptoms and the Global Functioning Scale (GFS)^3^ to assess general functioning (Supplementary Table S2).

| **Supplementary Table S2. Clinical measures** | |
| --- | --- |
| **Structured Interview for Prodromal Syndromes/Scale of Prodromal Symptoms** | |
| **Positive Symptoms** |  |
| P1 – Unusual thought content/Delusional ideas | Symptom score ranges from 0-6 |
| P2 – Suspiciousness/Persecutory ideas | Symptom score ranges from 0-6 |
| P3 – Grandiose ideas | Symptom score ranges from 0-6 |
| P4 – Perceptual abnormalities/Hallucinations | Symptom score ranges from 0-6 |
| P5 – Disorganized communication | Symptom score ranges from 0-6 |
| **Negative Symptoms** |  |
| N1 – Social isolation or withdrawal | Symptom score ranges from 0-6 |
| N2 – Avolition | Symptom score ranges from 0-6 |
| N3 – Decreased expression of emotion | Symptom score ranges from 0-6 |
| N4 – Decreased experience of emotion | Symptom score ranges from 0-6 |
| N5 – Decreased ideational richness | Symptom score ranges from 0-6 |
| N6 – Deterioration of occupational functioning | Symptom score ranges from 0-6 |
| **Disorganization Symptoms** |  |
| D1 – Odd behavior or appearance | Symptom score ranges from 0-6 |
| D2 – Bizarre thinking | Symptom score ranges from 0-6 |
| D3 – Trouble with focus and attention | Symptom score ranges from 0-6 |
| D4 – Impairment in personal hygiene | Symptom score ranges from 0-6 |
| **General Symptoms** |  |
| G1 – Sleep disturbance | Symptom score ranges from 0-6 |
| G2 – Dysphoric mood | Symptom score ranges from 0-6 |
| G3 – Motor disturbance | Symptom score ranges from 0-6 |
| G4 – impaired tolerance to stress | Symptom score ranges from 0-6 |
| **Global Functioning Scale** | |
| Social | Functioning score ranges from 1-10 |
| Role | Functioning score ranges from 1-10 |

- - 1. **Language assessment**

Language assessment was undertaken in a quiet, private room. Interviews took place at Columbia University, New York and at the Icahn School of Medicine at Mount Sinai (ISMMS). Open-ended, narrative interviews of ~30-45 minutes were obtained from participants by interviewers trained by an expert in qualitative interviewing and phenomenological research^4^. All interviews were prompted with the question, “How have things been going for you lately”. If necessary, participants may have been probed into the past or future. Speech samples were audio-recorded with a Snowball microphone by Blue (<https://www.bluedesigns.com/>) using Audacity (https://www.audacityteam.org/). Following the interview, the recorded speech samples were transferred to local on-site computers and transcribed by an independent company (<https://sftp.transcribeme.com>). Subsequently, speech samples were manually examined and deidentified of any personal or identifying information by trained research assistants.

- - 1. **Speech Processing**

The language features were computed from the speech samples using the Natural Language Toolkit (NLTK)^5^. Content from the interviewer were removed from all speech samples. First, individual words were identified in the text, and punctuation marks were discarded, resulting in a list of words for each text, with repetitions. Each interview was then parsed into sentences, and the parts of speech (e.g., nouns) were identified using the Treebank tagger supplied by NLTK. Each word was lemmatized using the WordNet lemmatizer from NLTK: which corresponds to converting words into the root from which they are inflected. Previous studies have shown that word lemmatizing facilitates robust measurement of abstract concepts and topological features in texts^6,7^. Preprocessing resulted in a list of lemmatized words, each one in a new line maintaining original order, in lowercase and without punctuation marks or symbols. Each interview thus resulted in a string of N tokens {wi}={w1,w2,…,wN} to be use for the semantic and structural analyzers.

- - 1. **Speech Analyses**

For semantic analyses, we used latent semantic analysis (LSA)^8,9^, a well-validated approach to automated text analysis previously used to analyze speech in schizophrenia^10^ and CHR^11^. LSA was used to convert each transcript from a series of words into a series of semantic vectors, maintaining the original order of the transcribed text. In this analysis, a high‐dimensional semantic vector is assigned to each word in the lexicon based on its co‐occurrence with other words in a very large corpus of text, specifically the Touchstone Applied Science Associates (TASA) corpus, a collection of educational materials.

Automated analysis provides a construction of meaning in language that resembles what the human mind does, i.e. to learn the meaning of words in terms of prior experience with those words in different contexts. The computer “learns” the meaning of words computationally, by scanning a very large corpus of text and determining the frequency of co‐occurrence of each word with every other word in the lexicon. Words that co‐occur more frequently are considered to have greater semantic similarity (e.g., “cat”/“dog” vs. “cat”/“pencil”), and the direction of their vectors will be more aligned. Aggregates of words (e.g., sentences) have semantic vectors that are the sum of semantic vectors for all the words they contain. Semantic coherence between words, or between aggregates (e.g., successive sentences), can be indexed by calculating the cosine between successive semantic vectors (from −1.0 for incoherence to 1.0 for coherence). As in our prior studies^11,12^, we calculated typical statistical measures of coherence, such as mean, standard deviation, minimum, and maximum, “normalized” or adjusted for sentence length. Similarly, we calculated statistical measures of sentence length including mean, median, standard deviation, minimum, and maximum values. As the measures for semantic coherence did not include the median, we excluded median sentence length in all subsequent analyses.

Just as each word in every transcript was assigned a semantic vector, each word was also tagged in respect to its grammatical function, using the POS‐Tag procedures in the open‐access Natural Language Toolkit (www.nltk.org) in reference to a hand‐tagged corpus called the Penn Treebank^5^. For example, the sentence “The dog is near the fence” would be tagged as (“The”, “DT”), (“dog”, “NN”), (“is”, “VBZ”), (“near”, “IN”), (“the”, “DT”), (“fence”, “NN”), where DT is the tag for determiners, NN for nouns, VBZ for verbs, and IN for prepositions. The Penn Treebank has thirty‐six part‐of‐speech tags, which include types of nouns, verbs, adjectives, adverbs, determiners, prepositions and pronouns.

- - 1. **Code availability**

Code for speech preprocessing (WordNet lemmatizer) and POS-Tag (Penn Tree Bank) is available open access through the NLTK (<http://www.nltk.org/>)^5^.

| **Supplementary Table S3. Definition of linguistic features extracted via Latent Semantic Analysis and Part Of Speech tagging using the Natural Language Toolkit (NLTK)** | |
| --- | --- |
| Amount of Speech | |
| Mean sentence length | NLTK tokenization first split sentences into words; the mean number of words was computed across all sentences. |
| Median sentence length | NLTK tokenization first split sentences into words; the mean number of words was computed across all sentences |
| Standard deviation sentence length | This is the standard deviation of sentence length. |
| Minimum sentence length | Following the process defined above, the minimum number of words was computed across all sentences. |
| Maximum sentence length | Following the process defined above the maximum number of words was computed across all sentences. |
| Sematic Properties | |
| Mean semantic coherence | Latent Semantic Analysis (LSA) was used to analyze sentence-to-sentence coherence. After separating the text into sentences, the vector for each sentence was computed (as the weighted sum of it its weighted terms) and was compared to the vector for the next sentence in the text. The cosine value of two vectors represents the semantic coherence between two sentences, ranging from −1.0 (incoherence) to 1.0 (coherence). We then computed the mean semantic coherence measures for all consecutive sentence pairs. |
| Standard deviation semantic coherence | This is the standard deviation of sentence-to-sentence semantic coherence as defined above. |
| Minimum semantic coherence | Following the process defined above we computed the minimum semantic coherence for all consecutive sentence pairs. |
| Maximum semantic coherence | Following the process defined above we computed the maximum semantic coherence for all consecutive sentence pairs |
| Parts of Speech Tagging |  |
| Coordinating conjunction (CC) | NLTK was used to label each word by its grammatical function based on Part of Speech tagging (POS-tag). The CC variable was computed as the sum of occurrences of this POS-tag in a given sentence, divided by its length and averaged across all sentences. |
| Cardinal number (CD) | NLTK was used to label each word by its grammatical function based on Part of Speech tagging (POS-tag). The CD variable was computed as the sum of occurrences of this POS-tag in a given sentence, divided by its length and averaged across all sentences. |
| Determiner (DT) | NLTK was used to label each word by its grammatical function based on Part of Speech tagging (POS-tag). The DT variable was computed as the sum of occurrences of this POS-tag in a given sentence, divided by its length and averaged across all sentences. |
| Existential *there* (EX) | NLTK was used to label each word by its grammatical function based on Part of Speech tagging (POS-tag). The EX variable was computed as the sum of occurrences of this POS-tag in a given sentence, divided by its length and averaged across all sentences. |
| Foreign word (FW) | NLTK was used to label each word by its grammatical function based on Part of Speech tagging (POS-tag). The FW variable was computed as the sum of occurrences of this POS-tag in a given sentence, divided by its length and averaged across all sentences. |
| Preposition or subordinating conjunction | NLTK was used to label each word by its grammatical function based on Part of Speech tagging (POS-tag). The preposition variable was computed as the sum of occurrences of this POS-tag in a given sentence, divided by its length and averaged across all sentences. |
| Adjective | NLTK was used to label each word by its grammatical function based on Part of Speech tagging (POS-tag). The adjective variable was computed as the sum of occurrences of this POS-tag in a given sentence, divided by its length and averaged across all sentences. |
| Adjective, comparative | NLTK was used to label each word by its grammatical function based on Part of Speech tagging (POS-tag). The comparative adjective variable was computed as the sum of occurrences of this POS-tag in a given sentence, divided by its length and averaged across all sentences. |
| Adjective, superlative | NLTK was used to label each word by its grammatical function based on Part of Speech tagging (POS-tag). The superlative adjective variable was computed as the sum of occurrences of this POS-tag in a given sentence, divided by its length and averaged across all sentences. |
| List item marker | NLTK was used to label each word by its grammatical function based on Part of Speech tagging (POS-tag). The list item marker variable was computed as the sum of occurrences of this POS-tag in a given sentence, divided by its length and averaged across all sentences. |
| Modal verb | NLTK was used to label each word by its grammatical function based on Part of Speech tagging (POS-tag). The modal verb variable was computed as the sum of occurrences of this POS-tag in a given sentence, divided by its length and averaged across all sentences. |
| Noun, singular or mass | NLTK was used to label each word by its grammatical function based on Part of Speech tagging (POS-tag). The singular or mass noun variable was computed as the sum of occurrences of this POS-tag in a given sentence, divided by its length and averaged across all sentences. |
| Noun, plural | NLTK was used to label each word by its grammatical function based on Part of Speech tagging (POS-tag). The plural noun variable was computed as the sum of occurrences of this POS-tag in a given sentence, divided by its length and averaged across all sentences. |
| Proper noun, singular | NLTK was used to label each word by its grammatical function based on Part of Speech tagging (POS-tag). The proper singular noun variable was computed as the sum of occurrences of this POS-tag in a given sentence, divided by its length and averaged across all sentences. |
| Proper noun, plural | NLTK was used to label each word by its grammatical function based on Part of Speech tagging (POS-tag). The proper plural noun variable was computed as the sum of occurrences of this POS-tag in a given sentence, divided by its length and averaged across all sentences. |
| Predeterminer | NLTK was used to label each word by its grammatical function based on Part of Speech tagging (POS-tag). The predeterminer variable was computed as the sum of occurrences of this POS-tag in a given sentence, divided by its length and averaged across all sentences. |
| Possessive ending | NLTK was used to label each word by its grammatical function based on Part of Speech tagging (POS-tag). The possessive ending variable was computed as the sum of occurrences of this POS-tag in a given sentence, divided by its length and averaged across all sentences. |
| Personal pronoun | NLTK was used to label each word by its grammatical function based on Part of Speech tagging (POS-tag). The personal pronoun variable was computed as the sum of occurrences of this POS-tag in a given sentence, divided by its length and averaged across all sentences. |
| Possessive pronoun | NLTK was used to label each word by its grammatical function based on Part of Speech tagging (POS-tag). The possesive pronoun variable was computed as the sum of occurrences of this POS-tag in a given sentence, divided by its length and averaged across all sentences. |
| Adverb | NLTK was used to label each word by its grammatical function based on Part of Speech tagging (POS-tag). The adverb variable was computed as the sum of occurrences of this POS-tag in a given sentence, divided by its length and averaged across all sentences. |
| Adverb, comparative | NLTK was used to label each word by its grammatical function based on Part of Speech tagging (POS-tag). The comparative adverb variable was computed as the sum of occurrences of this POS-tag in a given sentence, divided by its length and averaged across all sentences. |
| Adverb, superlative | NLTK was used to label each word by its grammatical function based on Part of Speech tagging (POS-tag). The superlative adverb variable was computed as the sum of occurrences of this POS-tag in a given sentence, divided by its length and averaged across all sentences. |
| Particle | NLTK was used to label each word by its grammatical function based on Part of Speech tagging (POS-tag). The particle variable was computed as the sum of occurrences of this POS-tag in a given sentence, divided by its length and averaged across all sentences. |
| *“To”* | NLTK was used to label each word by its grammatical function based on Part of Speech tagging (POS-tag). The “To” variable was computed as the sum of occurrences of this POS-tag in a given sentence, divided by its length and averaged across all sentences. |
| Interjection | NLTK was used to label each word by its grammatical function based on Part of Speech tagging (POS-tag). The interjection variable was computed as the sum of occurrences of this POS-tag in a given sentence, divided by its length and averaged across all sentences. |
| Verb, base form | NLTK was used to label each word by its grammatical function based on Part of Speech tagging (POS-tag). The base form verb variable was computed as the sum of occurrences of this POS-tag in a given sentence, divided by its length and averaged across all sentences. |
| Verb, past tense | NLTK was used to label each word by its grammatical function based on Part of Speech tagging (POS-tag). The past tense verb variable was computed as the sum of occurrences of this POS-tag in a given sentence, divided by its length and averaged across all sentences. |
| Verb, gerund or present participle | NLTK was used to label each word by its grammatical function based on Part of Speech tagging (POS-tag). The gerund/present particle verb variable was computed as the sum of occurrences of this POS-tag in a given sentence, divided by its length and averaged across all sentences. |
| Verb, past participle | NLTK was used to label each word by its grammatical function based on Part of Speech tagging (POS-tag). The past particle verb variable was computed as the sum of occurrences of this POS-tag in a given sentence, divided by its length and averaged across all sentences. |
| Verb, non-3^rd^ person singular present | NLTK was used to label each word by its grammatical function based on Part of Speech tagging (POS-tag). The non-3^rd^ person singular present verb variable was computed as the sum of occurrences of this POS-tag in a given sentence, divided by its length and averaged across all sentences. |
| Verb, 3^rd^ person singular present | NLTK was used to label each word by its grammatical function based on Part of Speech tagging (POS-tag). The 3rd person singular present verb variable was computed as the sum of occurrences of this POS-tag in a given sentence, divided by its length and averaged across all sentences. |
| Wh-determiner | NLTK was used to label each word by its grammatical function based on Part of Speech tagging (POS-tag). The Wh-determiner variable was computed as the sum of occurrences of this POS-tag in a given sentence, divided by its length and averaged across all sentences. |
| Wh-pronoun | NLTK was used to label each word by its grammatical function based on Part of Speech tagging (POS-tag). The Wh-pronoun variable was computed as the sum of occurrences of this POS-tag in a given sentence, divided by its length and averaged across all sentences. |
| Possessive wh-pronoun | NLTK was used to label each word by its grammatical function based on Part of Speech tagging (POS-tag). The possessive Wh-pronoun variable was computed as the sum of occurrences of this POS-tag in a given sentence, divided by its length and averaged across all sentences. |
| Wh-adverb | NLTK was used to label each word by its grammatical function based on Part of Speech tagging (POS-tag). The Wh-adverb variable was computed as the sum of occurrences of this POS-tag in a given sentence, divided by its length and averaged across all sentences. |

**1.2 Neuroimaging**

***1.2.1 Neuroimaging Acquisition***

*Columbia University***:** Participants were scanned at the MRI Research Unit at Columbia University Medical Center using a GE MR750 3T scanner (GE Medical Systems, Milwaukee, WI) with a 32-channel receiver coil. Structural data were acquired using the following parameters: Field of View (FOV): 240x240 mm, matrix size: 300×300, 0.8 mm isotropic resolution, Repetition Time (TR)= 2500 ms, Time to Echo (TE)=3.1 ms, Inversion Time (TI)=450 ms, 12 degree flip angle, total acquisition time (TA) = 7 min 30 sec. Resting-state functional data were acquired using the following parameters with eyes open, fixating on a central cross: 394 volumes, TE/TR = 25/850 ms, 2.0 mm isotropic resolution, no gap, 11 slices for whole brain coverage, FOV: 192x192 mm, matrix size: 96×96, 60 degree flip angle, TA = 5 min 35 sec.

*ISMMS:* All participants were scanned at the Leon and Norma Hess Center for Science and Medicine using a 3T Siemens Skyra scanner (Siemens Medical Systems, Erlangen, Germany) with a 32 channel receiver coil. Structural data were acquired using the following parameters: FOV: 256×256 mm, matrix size: 320×320, 0.8 mm isotropic resolution, TR = 2400 ms, TE = 2.07 ms, TI = 1000 ms, 8 degree flip angle, TA = 7 min 2 sec. Resting-state functional data were acquired with eyes fixating on a central cross. A single shot echo planar gradient echo T2* imaging sequence was used for the resting-state scan with eyes open, fixating on a central cross with the following parameters: 394 volumes, TE/TR=40.6/850 ms, 2.1 mm isotropic resolution, no gap, 54 slices for whole brain coverage, FOV: 192×192 mm, matrix size: 92×92, 60 degree-flip angle, and TA: 5 min 43 sec.

***1.2.2* Resting-state Functional MRI data processing**

All rs-fMRI data were preprocessed using the Statistical Parametric Mapping (SPM) 12 Toolbox (https://www.fil.ion.ucl.ac.uk/spm/software/spm12/) version 7487. The preprocessing included the following steps: discarding of the first 3 volumes, motion correction to the first volume with rigid-body alignment; coregistration between the functional scans and the anatomical T_1_ scan; spatial normalization of the functional images into MNI stereotaxic standard space; spatial smoothing with a 6-mm at full-width at half-maximum Gaussian kernel. Data were additionally preprocessed to correct for head motion using the following steps: wavelet despiking (removing signal transients related to small amplitude (<1 mm) head movements)^13^; detrending; and multiple regression of motion parameters and their derivatives (24-parameter model)^14^, as well as white matter (WM), cerebro-spinal fluid (CSF) time series and their linear trends. Lastly, a bandpass filter (0.01-0.08Hz) was applied^15^.

The time course of head motion was obtained by estimating the translations in each direction and the rotations in angular motion about each axis for each volume. Framewise displacement (FD), which indexes the volume-to-volume changes in head position, was calculated for each subject^16^. FD for the first volume of a run is by convention zero. Subjects with a mean FD of > 0.50 mm were excluded from further analyses.

Resting-state networks were defined in each participant using the functional templates available through the Functional Imaging in Neuropsychiatric Disorders Lab at Stanford University, USA (https://findlab.stanford.edu/functional_ROIs.html) (Supplementary Figure S1)^17^. We specifically examined the executive control network (ECN), the salience network (SAL), the somatosensory network (SMN), the auditory network (AN), and the language network (LAN). For these analyses, the sub-divisions of the DMN [dorsal DMN (dDMN), ventral DMN (vDMN), the precuneus network (PN)] were merged into a single network. The left and right ECN were merged into a single bilateral network, and the anterior and posterior SAL were also merged to form a single network. Fisher Z-transformed Pearson’s correlation coefficients were computed to calculate the cohesiveness of each network (i.e., within-network functional connectivity) and the integration of the LAN (i.e., between network connectivity) with the DMN, ECN, SAL, SMN and AN networks. Cohesiveness was computed as the average correlation of each voxel’s blood-oxygen-dependent-level (BOLD) signal time series with every other gray matter voxel within each network. Integration was computed as the correlation between the average time-series of each pair of networks. These computations yielded 11 connectivity measures which are shown in Supplementary Table S4.

Figure S1: Spatial distribution of the resting-state networks and subnetworks based on the functional templates available through the Functional Imaging in Neuropsychiatric Disorders Lab at Stanford University, USA^17^.


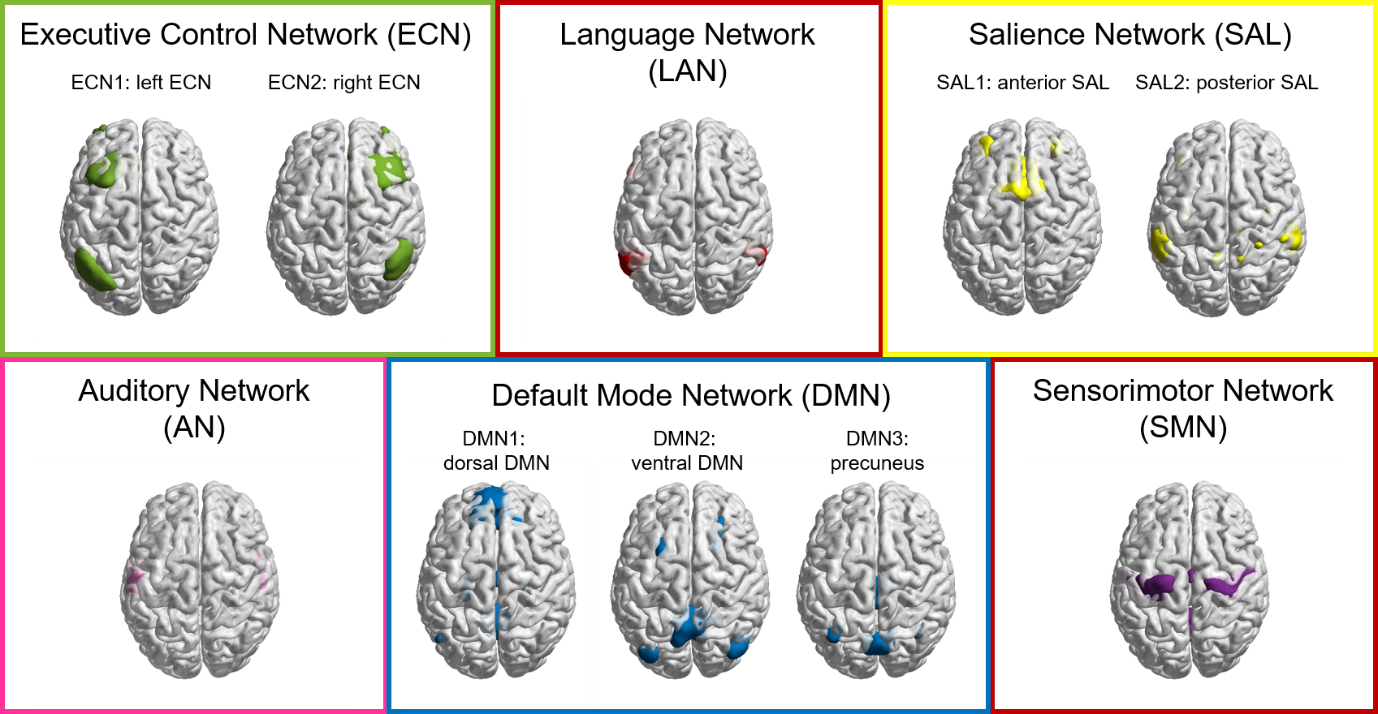


| **Supplementary Table S4. Definition functional imaging measures; all networks were defined using parcellation templates provided by** **Shirer et al, 2012** | |
| --- | --- |
| LAN-Cohesiveness | Within-network connectivity of the language network (LAN) network |
| DMN-Cohesiveness | Within-network connectivity of the default-mode network (DMN) |
| ECN-Cohesiveness | Within-network connectivity of the executive control network (ECN) |
| SAL-Cohesiveness | Within-network connectivity of the salience network (SAL) |
| SMN-Cohesiveness | Within-network connectivity of the sensorimotor network (SMN) |
| AN-Cohesiveness | Within-network connectivity of the auditory network (AN) |
| LAN-DMN | Integration (i.e., between-network connectivity) between the language (LAN) and the default-mode network (DMN) |
| LAN-ECN | Integration (i.e., between-network connectivity) between the language (LAN) and the executive control network (ECN) |
| LAN-SAL | Integration (i.e., between-network connectivity) between the language (LAN) and the salience network (SAL) |
| LAN-SMN | Integration (i.e., between-network connectivity) between the language (LAN) and the sensorimotor network (SMN) |
| LAN-AN | Integration (i.e., between-network connectivity) between the language (LAN) and the auditory network (AN) |

**1.2.3 *Structural MRI data processing***

Structural imaging data were analysed using FreeSurfer 6.0 (http://surfer.nmr.mgh.harvard.edu/). The steps included removal of non-brain tissue using a hybrid watershed/surface deformation procedure^18^, automated Talairach transformation, segmentation of the subcortical white matter and deep gray matter volumetric structures^19,20^ intensity normalization^21^, tessellation of the boundary between the gray and white matter, automated topology correction^22,23^, and surface deformation following intensity gradients to optimally place the gray/white matter boundaries and gray/cerebrospinal fluid borders at the location where the greatest shift in intensity defines the transition to the other tissue class. Subsequently, subcortical volume and of cortical thickness (calculated as the closest distance from the gray/white boundary to the gray/CSF boundary at each vertex on the tessellated surface) measures were extracted. . This process yielded 68 cortical thickness measures and 20 subcortical volume measures (Supplementary Table S5). Prior to being entered into further analyses, subcortical volumes were adjusted for variation in intracranial volume (ICV) in accordance to Pintzka et al.^24^ using the following equation: ${Vol}_{adj}=Vol- \beta*(ICV- \bar{ICV})$, where *Vol_adj_* is the ICV-adjusted volume, *Vol* is the original uncorrected volume, *β* is the slope from the linear regression of Vol on ICV, ICV is the ICV of a study participant and $\bar{ICV}$ is the mean ICV across all study participants. The same approach was used for correcting for cortical thickness using mean cortical thickness for each hemisphere.

| **Supplementary Table S5. Definition of the morphometric measures derived from Freesurfer 6.0** | |
| --- | --- |
| **Cortical Thickness** | |
| Banks superior temporal gyrus | left and right measures were considered separately |
| Caudal anterior cingulate cortex | left and right measures were considered separately |
| Caudal middle frontal gyrus | left and right measures were considered separately |
| Cuneus | left and right measures were considered separately |
| Entorhinal cortex | left and right measures were considered separately |
| Fusiform gyrus | left and right measures were considered separately |
| Inferior parietal lobule | left and right measures were considered separately |
| Inferior temporal gyrus | left and right measures were considered separately |
| Isthmus cingulate cortex | left and right measures were considered separately |
| Lateral occipital gyrus | left and right measures were considered separately |
| Lateral orbitofrontal gyrus | left and right measures were considered separately |
| Lingual gyrus | left and right measures were considered separately |
| Medial orbitofrontal gyrus | left and right measures were considered separately |
| Middle temporal gyrus | left and right measures were considered separately |
| Parahippocampal gyrus | left and right measures were considered separately |
| Paracentral gyrus | left and right measures were considered separately |
| Pars opercularis | left and right measures were considered separately |
| Pars orbitalis | left and right measures were considered separately |
| Pars triangularis | left and right measures were considered separately |
| Pericalcarine gyrus | left and right measures were considered separately |
| Postcentral gyrus | left and right measures were considered separately |
| Posterior cingulate cortex | left and right measures were considered separately |
| Precentral gyrus | left and right measures were considered separately |
| Precuneus | left and right measures were considered separately |
| Rostral anterior cingulate cortex | left and right measures were considered separately |
| Rostral middle frontal gyrus | left and right measures were considered separately |
| Superior frontal gyrus | left and right measures were considered separately |
| Superior parietal lobule | left and right measures were considered separately |
| Superior temporal gyrus | left and right measures were considered separately |
| Supramarginal gyrus | left and right measures were considered separately |
| Frontal pole | left and right measures were considered separately |
| Temporal pole | left and right measures were considered separately |
| Transverse temporal gyrus | left and right measures were considered separately |
| Insula | left and right measures were considered separately |
| **Subcortical Volumes** | |
| Thalamus | left and right measures were considered separately following ICV correction |
| Hippocampus | left and right measures were considered separately following ICV correction |
| Caudate nucleus | left and right measures were considered separately following ICV correction |
| Nucleus Accumbens | left and right measures were considered separately following ICV correction |
| Pallidum | left and right measures were considered separately following ICV correction |
| Putamen | left and right measures were considered separately following ICV correction |
| Amygdala | left and right measures were considered separately following ICV correction |
| Ventral diencephalon | left and right measures were considered separately following ICV correction |
| Cerebellum | left and right measures were considered separately following ICV correction |
| Lateral ventricles | left and right measures were considered separately following ICV correction |

- 1. **Case-Control differences in structural and functional connectivity**

All imaging data were harmonized for site using ComBat. Group differences in brain structure and connectivity were assessed after regressing the effect of age and sex. Differences were considered significant at p<0.05 using FDR-correction, and were assessed at the level of the total sample and within each site.

- 1. **Sparse Canonical Correlation Analysis**

Sparse Canonical correlation analysis (sCCA)^25^ is a multivariate approach that acts as dimensionality reduction technique, reducing each dataset to a number of linear combinations of canonical variables also known as canonical variates. Each pair of canonical variates (or modes) are calculated so that the correlations are maximized between them. The steps involved in each sCCA performed were as follows:

(1) Residualization of imaging, clinical, and language measures for age and sex for all participants; the language and clinical measures were also residualised for site while the imaging measures were harmonized for site with ComBat^26^. For the sCCA model that included the rsfMRI data, data were also residualised for head motion based on mean framewise displacement.

(2) Data were normalized by calculating Z-scores for each variable;

(3) sCCA models were implemented in MatlabR2018b using an in-house script according to our previously published work^27-29^, to identify the linear combination of variables in each dataset in each model (i.e., language-clinical features; language-functional connectivity and language-brain structure);

(4) Calculation of exact P-values for canonical correlations were based on 10000 randomly permuted datasets;

(5) False Discovery Rate (FDR) correction^30^ was used to control for testing of multiple variates and a p<0.05 was considered statistically significant.

**2. Supplementary Results**

**2.1 Language**

| **Supplementary Table S6. Linguistic characteristics of the sample recruited at each site** | | | | |
| --- | --- | --- | --- | --- |
|  | **Columbia University** | | **ISMMS** | |
| **Linguistic Variable** | **CHR**  **Individuals**  **N=17** | **Healthy**  **individuals**  **N=13** | **CHR Individuals**  **N=29** | **Healthy individuals**  **N=9** |
| Amount of Speech |  |  |  |  |
| Sentence length, mean | 13.71 (3.18) | 12.23 (2.86) | 13.41 (2.69) | 14.44 (4.98) |
| Sentence length, standard deviation | 10.65 (3.22) | 9.31 (2.18) | 9.9 (2.14) | 11.11 (3.55) |
| Sentence length, maximum | 68.71 (23.3) | 66.23 (17.22) | 60.34 (13.79) | 67.78 (13.87) |
| Semantic Properties |  |  |  |  |
| Semantic coherence, mean | 0.81 (0.04) | 0.78 (0.05) | 0.81 (0.05) | 0.8 (0.04) |
| Semantic coherence, standard deviation | 0.18 (0.03) | 0.19 (0.03) | 0.18 (0.04) | 0.18 (0.02) |
| Semantic coherence, minimum | -0.06 (0.16) | -0.09 (0.16) | 0.07 (0.18) | 0.046 (0.15) |
| Semantic coherence, maximum | 0.99 (0.01) | 1 (0.01) | 0.99 (0.01) | 1 (0.005) |
| Parts of Speech-Tagging |  |  |  |  |
| Coordinating conjunction | 0.03 (0.01) | 12.23 (2.86) | 0.04 (0.01) | 0.03 (0.01) |
| Cardinal number | 0.004 (0.003) | 9.31 (2.18) | 0.01 (0.004) | 0.01 (0.002) |
| Determiner | 0.06 (0.01) | 66.23 (17.22) | 0.06 (0.01) | 0.06 (0.01) |
| Existential *there* | 0.002 (0.001) | 0.78 (0.05) | 0.002 (0.001) | 0.003 (0.001) |
| Preposition or subordinating conjunction | 0.07 (0.01) | 0.19 (0.03) | 0.07 (0.01) | 0.07 (0.01) |
| Adjective | 0.04 (0.01) | -0.09 (0.16) | 0.04 (0.01) | 0.04 (0.01) |
| Adjective, comparative | 0.002 (0.001) | 1 (0.01) | 0.002 (0.002) | 0.002 (0.001) |
| Adjective, superlative | 0.001 (0.001) | 0.03 (0.01) | 0.001 (0.001) | 0.001 (0.0005) |
| Modal verb | 0.01 (0.005) | 0.01 (0.004) | 0.01 (0.003) | 0.01 (0.003) |
| Noun, singular or mass | 0.08 (0.01) | 0.06 (0.005) | 0.08 (0.01) | 0.08 (0.01) |
| Noun, plural | 0.02 (0.01) | 0.002 (0.001) | 0.02 (0.005) | 0.02 (0.005) |
| Proper noun, singular | 0.02 (0.01) | 0.06 (0.01) | 0.017 (0.01) | 0.02 (0.01) |
| Predeterminer | 0.001 (0.001) | 0.04 (0.01) | 0.001 (0.001) | 0.001 (0.001) |
| Possessive ending | 0.0003 (0.0003) | 0.002 (0.001) | 0.0003 (0.0003) | 0.0004 (0.001) |
| Personal pronoun | 0.11 (0.01) | 0.001 (0.001) | 0.11 (0.01) | 0.1 (0.01) |
| Possessive pronoun | 0.01 (0.003) | 0.01 (0.003) | 0.01 (0.003) | 0.01 (0.003) |
| Adverb | 0.08 (0.01) | 0.09 (0.01) | 0.09 (0.01) | 0.08 (0.01) |
| Adverb, comparative | 0.002 (0.001) | 0.02 (0.01) | 0.002 (0.001) | 0.002 (0.001) |
| Adverb, superlative | 0.0001 (0.0002) | 0.03 (0.01) | 0.0003 (0.0003) | 0.0002 (0.0003) |
| Particle | 0.003 (0.001) | 0.001 (0.001) | 0.004 (0.001) | 0.004 (0.001) |
| *“To”* | 0.02 (0.003) | 0.001 (0.0004) | 0.02 (0.004) | 0.02 (0.003) |
| Interjection | 0.04 (0.02) | 0.11 (0.01) | 0.04 (0.02) | 0.05 (0.03) |
| Verb, base form | 0.03 (0.01) | 0.01 (0.003) | 0.04 (0.01) | 0.04 (0.01) |
| Verb, past tense | 0.03 (0.01) | 0.08 (0.01) | 0.03 (0.01) | 0.03 (0.01) |
| Verb, gerund or present participle | 0.02 (0.004) | 0.001 (0.001) | 0.018 (0.01) | 0.02 (0.004) |
| Verb, past participle | 0.01 (0.002) | 0.0002 (0.0002) | 0.01 (0.003) | 0.01 (0.003) |
| Verb, non-3^rd^ person singular present | 0.05 (0.01) | 0.004 (0.002) | 0.05 (0.01) | 0.04 (0.01) |
| Verb, 3rd person singular present | 0.03 (0.01) | 0.02 (0.003) | 0.03 (0.01) | 0.03 (0.01) |
| Wh-determiner | 0.003 (0.002) | 0.05 (0.03) | 0.002 (0.001) | 0.004 (0.002) |
| Wh-pronoun | 0.01 (0.003) | 0.03 (0.01) | 0.01 (0.003) | 0.01 (0.002) |
| Wh-adverb | 0.01 (0.002) | 0.03 (0.01) | 0.01 (0.002) | 0.005 (0.001) |
| Continuous variables are shown as mean (standard deviation); CHR=Clinical high risk | | | | |

**2.2 sCCA for language-and clinical features**

This analysis identified a single significant mode as described in the main text. The best sparsity parameters were 0.5 for the clinical dataset and 0.8 for the language dataset. Supplementary Tables S7 and S8 show the weights of all the variables considered in the sCCA model.

| **Supplementary Table S7. Weights of the linguistic features in the significant mode of the sparse canonical correlation of linguistic and clinical measures in the CHR sample** | |
| --- | --- |
| **Amount of Speech** | |
| Sentence length, mean | 0.13 |
| Sentence length, standard deviation | 0.17 |
| Sentence length, maximum | 0.13 |
| **Sematic Properties** | |
| Semantic coherence, mean | 0.03 |
| Semantic coherence, standard deviation | -0.11 |
| Semantic coherence, minimum | 0.16 |
| Semantic coherence, maximum | -0.04 |
| **Parts of Speech Tagging** | |
| Coordinating conjunction | 0.23 |
| Cardinal number | -0.04 |
| Determiner | -0.18 |
| Existential *there* | -0.30 |
| Preposition or subordinating conjunction | 0.06 |
| Adjective | 0.02 |
| Adjective, comparative | 0.02 |
| Adjective, superlative | 0.07 |
| Modal verb | -0.26 |
| Noun, singular or mass | 0.01 |
| Noun, plural | -0.09 |
| Proper noun, singular | -0.04 |
| Predeterminer | 0.10 |
| Possessive ending | -0.03 |
| Personal pronoun | -0.17 |
| Possessive pronoun | 0.14 |
| Adverb | 0.22 |
| Adverb, comparative | -0.01 |
| Adverb, superlative | -0.13 |
| Particle | -0.10 |
| *“To”* | 0 |
| Interjection | 0.09 |
| Verb, base form | -0.16 |
| Verb, past tense | 0.23 |
| Verb, gerund or present participle | 0.17 |
| Verb, past participle | 0.30 |
| Verb, non-3^rd^ person singular present | -0.35 |
| Verb, 3^rd^ person singular present | -0.09 |
| Wh-determiner | 0.09 |
| Wh-pronoun | -0.39 |
| Wh-adverb | -0.08 |

| **Supplementary Table S8. Weights of the clinical features in the significant mode of the sparse canonical correlation of linguistic and clinical measures in the CHR sample** | |
| --- | --- |
| **Structured Interview for Prodromal Syndromes/Scale of Prodromal Symptoms** | |
| **Positive Symptoms** | 0 |
| P1 – Unusual thought content | 0 |
| P2 – Suspiciousness | 0 |
| P3 – Grandiosity | 0 |
| P4 – Perceptual abnormalities | 0 |
| P5 – Conceptual disorganization | 0 |
| **Negative Symptoms** |  |
| N1 – Social isolation or withdrawal | 0 |
| N2 – Avolition | 0.35 |
| N3 – Decreased expression of emotion | 0 |
| N4 – Decreased experience of emotion | 0.28 |
| N5 – Decreased ideational richness | -0.35 |
| N6 – Deterioration of role functioning | 0 |
| **Disorganization Symptoms** |  |
| D1 – Odd behavior or appearance | 0 |
| D2 – Bizarre thinking | -0.62 |
| D3 – Trouble with focus and attention | 0 |
| D4 – Impairment in personal hygiene | 0 |
| **General Symptoms** |  |
| G1 – Sleep disturbance | 0.12 |
| G2 – Dysphoric mood | 0.06 |
| G3 – Motor disturbance | 0 |
| G4 – impaired tolerance to stress | 0.52 |
| **Global Functioning Scale** | |
| Social | 0 |
| Role | 0 |

**2.3 sCCA for language-and resting-state network connectivity**

This analysis identified a single significant mode as described in the main text. The best sparsity parameters were 0.7 for the functional imaging dataset and 0.6 for the language dataset. Supplementary Tables S9 and S10 show the weights of all the variables considered in the sCCA model.

| **Supplementary Table S9. Weights of the linguistic features in the significant mode of the sparse canonical correlation of linguistic and brain functional measures in the whole sample** | |
| --- | --- |
| Amount of Speech | |
| Sentence length, mean | 0 |
| Sentence length, standard deviation | 0 |
| Sentence length, maximum | 0 |
| Sematic Properties | |
| Semantic coherence, mean | 0 |
| Semantic coherence, standard deviation | 0 |
| Semantic coherence, minimum | 0 |
| Semantic coherence, maximum | 0.53 |
| Parts of Speech Tagging | |
| Coordinating conjunction | -0.10 |
| Cardinal number | -0.16 |
| Determiner | 0.09 |
| Existential *there* | 0 |
| Preposition or subordinating conjunction | 0 |
| Adjective | 0.11 |
| Adjective, comparative | 0.10 |
| Adjective, superlative | -0.47 |
| Modal verb | 0 |
| Noun, singular or mass | -0.032 |
| Noun, plural | 0 |
| Proper noun, singular | -0.09 |
| Predeterminer | -0.14 |
| Possessive ending | -0.26 |
| Personal pronoun | 0 |
| Possessive pronoun | -0.22 |
| Adverb | -0.06 |
| Adverb, comparative | 0.25 |
| Adverb, superlative | 0 |
| Particle | 0 |
| *“To”* | 0 |
| Interjection | 0.23 |
| Verb, base form | -0.13 |
| Verb, past tense | -0.01 |
| Verb, gerund or present participle | -0.23 |
| Verb, past participle | 0 |
| Verb, non-3^rd^ person singular present | 0.12 |
| Verb, 3^rd^ person singular present | 0 |
| Wh-determiner | -0.28 |
| Wh-pronoun | 0.03 |
| Wh-adverb | -0.07 |

| **Supplementary Table S10. Weights of the functional features in the significant mode of the sparse canonical correlation of linguistic and brain functional measures in the whole sample** | |
| --- | --- |
| LAN Cohesiveness | 0.57 |
| DMN Cohesiveness | 0.17 |
| ECN Cohesiveness | 0.64 |
| SAL Cohesiveness | 0.37 |
| SMN Cohesiveness | 0 |
| AN Cohesiveness | 0.19 |
| LAN-DMN | 0 |
| LAN-ECN | 0 |
| LAN-SAL | 0.09 |
| LAN-SMN | 0 |
| LAN-AN | 0.21 |

**2.4 sCCA for language-and brain structure**

This analysis identified a single significant mode as described in the main text. The best sparsity parameters were 0.8 for both the structural imaging and language datasets. Supplementary Tables S11 and S12 show the weights of all the variables considered in the sCCA model.

| **Supplementary Table S11. Weights of the linguistic features in the significant mode of the sparse canonical correlation of linguistic and brain structural measures in the whole sample** | |
| --- | --- |
| Amount of Speech | |
| Mean sentence length | 0.34 |
| Standard deviation sentence length | 0.31 |
| Maximum sentence length | 0.25 |
| Sematic Properties | |
| Mean semantic coherence | 0.37 |
| Standard deviation semantic coherence | -0.25 |
| Minimum semantic coherence | 0.09 |
| Maximum semantic coherence | 0.08 |
| Parts of Speech Tagging |  |
| Coordinating conjunction | 0.18 |
| Cardinal number | -0.12 |
| Determiner | 0.20 |
| Existential *there* | 0.10 |
| Preposition or subordinating conjunction | 0.31 |
| Adjective | 0.09 |
| Adjective, comparative | 0.22 |
| Adjective, superlative | -0.06 |
| Modal verb | 0.08 |
| Noun, singular or mass | 0.10 |
| Noun, plural | -0.02 |
| Proper noun, singular | -0.20 |
| Predeterminer | -0.03 |
| Possessive ending | 0.00 |
| Personal pronoun | 0.07 |
| Possessive pronoun | 0.00 |
| Adverb | 0.13 |
| Adverb, comparative | -0.04 |
| Adverb, superlative | 0.08 |
| Particle | -0.04 |
| *“To”* | 0.17 |
| Interjection | -0.21 |
| Verb, base form | 0.06 |
| Verb, past tense | 0.00 |
| Verb, gerund or present participle | 0.10 |
| Verb, past participle | 0.17 |
| Verb, non-3^rd^ person singular present | 0.07 |
| Verb, 3^rd^ person singular present | 0.13 |
| Wh-determiner | 0.11 |
| Wh-pronoun | 0.14 |
| Wh-adverb | 0.01 |

| **Supplementary Table S12. Weights of the morphometric features in the significant mode of the sparse canonical correlation of linguistic and brain structural measures in the whole sample** | | |
| --- | --- | --- |
| **Cortical Thickness** | **Left hemisphere** | **Right hemisphere** |
| Banks superior temporal gyrus | -0.09 | 0.01 |
| Caudal anterior cingulate cortex | -0.07 | -0.09 |
| Caudal middle frontal gyrus | 0.02 | -0.06 |
| Cuneus | -0.06 | 0.03 |
| Entorhinal cortex | 0.06 | 0.08 |
| Fusiform gyrus | -0.02 | 0.10 |
| Inferior parietal lobule | -0.003 | 0.11 |
| Inferior temporal gyrus | 0.01 | 0.14 |
| Isthmus cingulate cortex | 0.05 | -0.05 |
| Lateral occipital gyrus | -0.05 | 0.01 |
| Lateral orbitofrontal gyrus | 0.15 | 0.02 |
| Lingual gyrus | -0.02 | -0.03 |
| Medial orbitofrontal gyrus | 0.09 | 0.17 |
| Middle temporal gyrus | 0.06 | 0.09 |
| Parahippocampal gyrus | -0.01 | -0.03 |
| Paracentral gyrus | -0.002 | 0.01 |
| Pars opercularis | 0.12 | 0.04 |
| Pars orbitalis | -0.02 | 0.02 |
| Pars triangularis | 0.11 | -0.05 |
| Pericalcarine gyrus | -0.09 | -0.07 |
| Postcentral gyrus | 0.04 | -0.01 |
| Posterior cingulate cortex | 0.03 | -0.07 |
| Precentral gyrus | 0.06 | 0.04 |
| Precuneus | -0.01 | -0.03 |
| Rostral anterior cingulate cortex | 0.13 | 0.18 |
| Rostral middle frontal gyrus | 0.04 | -0.06 |
| Superior frontal gyrus | 0.02 | 0.02 |
| Superior parietal lobule | -0.10 | -0.08 |
| Superior temporal gyrus | 0.12 | 0.13 |
| Supramarginal gyrus | -0.06 | 0.09 |
| Frontal pole | 0.08 | 0.21 |
| Temporal pole | 0.07 | 0.14 |
| Transverse temporal gyrus | 0.05 | 0.09 |
| Insula | -0.02 | -0.05 |
| **Subcortical Volumes** | | |
| Thalamus | -0.24 | -0.20 |
| Hippocampus | -0.10 | -0.15 |
| Caudate nucleus | -0.07 | -0.15 |
| Nucleus Accumbens | -0.27 | -0.25 |
| Pallidum | -0.20 | -0.14 |
| Putamen | -0.18 | -0.17 |
| Amygdala | -0.11 | -0.06 |
| Ventral diencephalon | -0.15 | -0.15 |

**2.5 sCCA for language and all neuroimaging features**

There were no significant modes for this analysis across all sparsity levels (*P* > 0.05).

**2.6 Case-Control differences in morphometry and functional connectivity**

*Brain Structure*: There were no significant case-control differences in brain morphometry at FDR corrected p<0.05 noted in 1) the entire sample, 2) the sample recruited at Columbia University and 3) the sample recruited at ISMMS.

*Functional Connectivity*: There were no significant case-control differences in brain functional connectivity at FDR corrected p<0.05 noted in 1) the entire sample, 2) the sample recruited at Columbia University and 3) the sample recruited at ISMMS.

**References**

1. First MB, Williams JB, Karg RS, Spitzer RL. Structured clinical interview for DSM-5—Research version (SCID-5 for DSM-5, research version; SCID-5-RV). Arlington, VA: American Psychiatric Association. 2015;1-94.
2. Miller TJ, McGlashan TH, Rosen JL, Cadenhead K, Ventura J, McFarlane W, et al. Prodromal assessment with the structured interview for prodromal syndromes and the scale of prodromal symptoms: predictive validity, interrater reliability, and training to reliability. Schizophrenia Bulletin. 2003;29(4):703-15.
3. Cornblatt BA, Auther AM, Niendam T, Smith CW, Zinberg J, Bearden CE, et al. Preliminary findings for two new measures of social and role functioning in the prodromal phase of schizophrenia. Schizophrenia Bulletin. 2007;33(3):688-702.
4. Davidson L. Phenomenological research in schizophrenia: From philosophical anthropology to empirical science. Journal of Phenomenological Psychology. 1994;25(1):104-30.
5. Bird S, Klein E, Loper E. Natural language processing with Python: analyzing text with the natural language toolkit. O'Reilly Media, Inc; 2009.
6. Diuk C, Fernandez Slezak D, Raskovsky I, Sigman M, Cecchi GA. A quantitative philology of introspection. Frontiers in Integrative Neuroscience. 2012;6:80.
7. Mota NB, Vasconcelos NA, Lemos N, Pieretti AC, Kinouchi O, Cecchi GA, Copelli M, Ribeiro S. Speech graphs provide a quantitative measure of thought disorder in psychosis. PloS One. 2012;7(4).
8. Landauer TK, Dumais ST. A solution to Plato's problem: The latent semantic analysis theory of acquisition, induction, and representation of knowledge. Psychological Review. 1997;104(2):211.
9. Landauer TK, Foltz PW, Laham D. An introduction to latent semantic analysis. Discourse Processes. 1998;25(2-3):259-84.
10. Elvevåg B, Foltz PW, Weinberger DR, Goldberg TE. Quantifying incoherence in speech: an automated methodology and novel application to schizophrenia. Schizophrenia Research. 2007;93(1-3):304-16.
11. Bedi G, Carrillo F, Cecchi GA, Slezak DF, Sigman M, Mota NB, Ribeiro S, Javitt DC, Copelli M, Corcoran CM. Automated analysis of free speech predicts psychosis onset in high-risk youths. NPJ Schizophrenia. 2015;1:15030.
12. Corcoran CM, Carrillo F, Fernández‐Slezak D, Bedi G, Klim C, Javitt DC, Bearden CE, Cecchi GA. Prediction of psychosis across protocols and risk cohorts using automated language analysis. World Psychiatry. 2018;17(1):67-75.
13. Patel AX, Kundu P, Rubinov M, Jones PS, Vértes PE, Ersche KD, Suckling J, Bullmore ET. A wavelet method for modeling and despiking motion artifacts from resting-state fMRI time series. Neuroimage. 2014;95:287-304.
14. Friston KJ, Williams S, Howard R, Frackowiak RS, Turner R. Movement‐related effects in fMRI time‐series. Magnetic Resonance in Medicine. 1996;35(3):346-55.
15. Cordes D, Haughton VM, Arfanakis K, Carew J, Turski PA, Moritz CH, Quigley MA, Meyerand ME. Frequencies contributing to functional connectivity in the cerebral cortex in "resting-state" data. AJNR. American Journal of Neuroradiology. 2001;22:1326-1333.
16. Power JD, Barnes KA, Snyder AZ, Schlaggar, BL, Petersen SE. Spurious but systematic correlations in functional connectivity MRI networks arise from subject motion. NeuroImage 2012;59, 2142-2154.
17. Shirer WR, Ryali S, Rykhlevskaia E, Menon V, Greicius MD. Decoding subject-driven cognitive states with whole-brain connectivity patterns. Cerebral Cortex. 2012;22(1):158-65.
18. Ségonne F, Dale AM, Busa E, Glessner M, Salat D, Hahn HK, Fischl B. A hybrid approach to the skull stripping problem in MRI. Neuroimage. 2004;22(3):1060-75.
19. Fischl B, Salat DH, Busa E, Albert M, Dieterich M, Haselgrove C, Van Der Kouwe A, Killiany R, Kennedy D, Klaveness S, Montillo A. Whole brain segmentation: automated labeling of neuroanatomical structures in the human brain. Neuron. 2002;33(3):341-55.
20. Fischl B, Salat DH, Van Der Kouwe AJ, Makris N, Ségonne F, Quinn BT, Dale AM. Sequence-independent segmentation of magnetic resonance images. Neuroimage. 2004;23:S69-84.
21. Sled JG, Zijdenbos AP, Evans AC. A nonparametric method for automatic correction of intensity nonuniformity in MRI data. IEEE Transactions on Medical Imaging. 1998;17(1):87-97.
22. Fischl B, Liu A, Dale AM. Automated manifold surgery: constructing geometrically accurate and topologically correct models of the human cerebral cortex. IEEE Transactions on Medical Imaging. 2001;20(1):70-80.
23. Ségonne F, Pacheco J, Fischl B. Geometrically accurate topology-correction of cortical surfaces using nonseparating loops. IEEE Transactions on Medical Imaging. 20072;26(4):518-29.
24. Pintzka CW, Hansen TI, Evensmoen HR, Håberg AK. Marked effects of intracranial volume correction methods on sex differences in neuroanatomical structures: a HUNT MRI study. Frontiers in Neuroscience. 2015;9:238.
25. Witten DM, Tibshirani R, Hastie T. A penalized matrix decomposition, with applications to sparse principal components and canonical correlation analysis. Biostatistics. 2009;10(3):515-34.
26. Fortin JP, Cullen N, Sheline YI, Taylor WD, Aselcioglu I, Cook PA, Adams P, Cooper C, Fava M, McGrath PJ, McInnis M. Harmonization of cortical thickness measurements across scanners and sites. Neuroimage. 2018;167:104-20.
27. Doucet GE, Moser DA, Luber MJ, Leibu E, Frangou S. Baseline brain structural and functional predictors of clinical outcome in the early course of schizophrenia. Molecular Psychiatry. 2018:1-0.
28. Moser DA, Doucet GE, Ing A, Dima D, Schumann G, Bilder RM, Frangou S. An integrated brain–behavior model for working memory. Molecular Psychiatry. 2018;23(10):1974-80.
29. Moser DA, Doucet GE, Lee WH, Rasgon A, Krinsky H, Leibu E, Ing A, Schumann G, Rasgon N, Frangou S. Multivariate associations among behavioral, clinical, and multimodal imaging phenotypes in patients with psychosis. JAMA Psychiatry. 2018;75(4):386-95.
30. Benjamini Y, Yekutieli D. The control of the false discovery rate in multiple testing under dependency. Annals of Statistics. 2001;1165-88.
